# 

**Published:** 1923-05

**Authors:** 


### HOSPITAL and HEALTH REVIEW

Established 1886 | No. 1862. Vol. LXXII.

New Series. Vol. II. No. 20 May 1923 Price Sixpence

CONTENTS.

| | PAGE | | PAGE |
|---|---|---|---|
| Sheltering the People | 191 | Hospital Progress and Finance | 204 |
| The Middle Classes in Hospital | 192 | The Voluntary Hospitals Commission | 205 |
| Notes and Comments | 193 | The Hospital and Nursing Exhibition | 206 |
| Tinkering with Tuberculosis | 197 | The Evolution of the Voluntary Hospital | 207 |
| A Local Committee urged to Continue | 198 | Correspondence | 210 |
| Jubilee of the Hospital Saturday Fund | 199 | The Hospital Association Conference | 211 |
| Panel Pay and Extended Services.—I. By R. W. Harris | 200 | Health Insurance Notes | 212 |
| Hospital Accounts and Finance. By Joseph E. Stone | 201 | Reviews of Books | 213 |
| A Conference on Day Nursery Schools | 202 | Echoes of the Month | 217 |
| Ministry of Health Estimates | 203 | Hospital and Health News | 219 |
| | | Public Health in Parliament | 220 |
| | | Recent Appointments | 220 |

